# Low sensitivity of the metabolic syndrome to identify adolescents with impaired glucose tolerance: an analysis of NHANES 1999–2010

**DOI:** 10.1186/1475-2840-13-83

**Published:** 2014-04-23

**Authors:** Mark D DeBoer, Matthew J Gurka

**Affiliations:** 1 Department of Pediatrics, University of Virginia, Charlottesville, Virginia 22908, USA; 2 Department of Biostatistics, School of Public Health, West Virginia University, Morgantown, WV 26506, USA

**Keywords:** Insulin resistance, Metabolic syndrome, Impaired glucose tolerance, Type 2 diabetes, Adolescents

## Abstract

**Background:**

The presence of impaired glucose tolerance (IGT) and metabolic syndrome (MetS) are two risk factors for Type 2 diabetes. The inter-relatedness of these factors among adolescents is unclear.

**Methods:**

We evaluated the sensitivity and specificity of MetS for identifying IGT in an unselected group of adolescents undergoing oral glucose tolerance tests (OGTT) in the National Health and Nutrition Evaluation Survey 1999–2010. We characterized IGT as a 2-hour glucose ≥140 mg/dL and MetS using ATP-III-based criteria and a continuous sex- and race/ethnicity-specific MetS Z-score at cut-offs of +1.0 and +0.75 standard deviations (SD) above the mean.

**Results:**

Among 1513 adolescents, IGT was present in 4.8%, while ATP-III-MetS was present in 7.9%. MetS performed poorly in identifying adolescents with IGT with a sensitivity/specificity of 23.7%/92.9% for ATP-III-MetS, 23.6%/90.8% for the MetS Z-score at +1.0 SD and 35.8%/85.0 for the MetS Z-score at +0.75 SD. Sensitivity was higher (and specificity lower) but was still overall poor among overweight/obese adolescents: 44.7%/83.0% for ATP-III-MetS, 43.1%/77.1% for the MetS Z-score at +1.0 SD and 64.3%/64.3% for MetS Z-score at +0.75 SD.

**Conclusion:**

This lack of overlap between MetS and IGT may indicate that assessment of MetS is not likely to be a good indicator of which adolescents to screen using OGTT. These data further underscore the importance of other potential contributors to IGT, including Type 1 diabetes and genetic causes of poor beta-cell function. Practitioners should keep these potential causes of IGT in mind, even when evaluating obese adolescents with IGT.

## Introduction

The persistent high prevalence of pediatric obesity has greatly increased risk of Type 2 diabetes mellitus (T2DM) in the current generation of children and adolescents, increasing the need for effective tools to predict those at highest risk [1,2]. One sensitive and specific marker of impending T2DM is impaired glucose tolerance (IGT), defined as a blood glucose (BG) ≥140 mg/dL at 2 hours after an oral glucola load as part of a fasting oral glucose tolerance test (OGTT) [3,4]. In a study of children and adolescents referred to an obesity treatment clinic, 24% of those with IGT at baseline progressed to have T2DM over a follow-up period of 20 +/− 10 months, while none of the children with normal glucose tolerance progressed to T2DM over the same time frame [5,6]. These findings are in contrast to a study of early adolescent European children that showed a high reversion to normal glucose tolerance over a 1-year period [7]. Nevertheless, IGT may also be an early marker of risk, with increased rate of IGT noted even among adolescents with rising fasting glucose levels in the normal range [8]. While OGTT’s are labor- and time-intensive tests, the ADA recognizes them as one option in screening for T2DM risk [9]. It should be noted, however, that IGT does not distinguish between risk of T2DM and early, pre-clinical signs of Type 1 diabetes mellitus (T1DM), which results in elevated post-glucola BG due to insufficient insulin secretion [10].

Another important potential tool to screen for risk of T2DM is a set of criteria to identify the metabolic syndrome (MetS), a cluster of cardiovascular risk factors that occur together more often than would be expected by chance [11]. These factors include central obesity, hypertension, hypertriglyceridemia, low HDL-cholesterol, and elevated fasting glucose [12] and show in increase in prevalence with age [13]. MetS appears to be associated with insulin resistance in adolescents in that increasing degrees of insulin resistance as determined by homeostasis model [14,15] and hyperinsulinemic clamp [16] are significantly associated with risk of MetS and/or its individual components. While the pathophysiology of T2DM consists of multiple overlying factors, including insulin resistance, excess hepatic glucose release and defects in adequate insulin production [17,18], the potential utility for MetS to identify risk in adolescents for future T2DM was demonstrated in that adolescents with MetS (compared to those without MetS) have an odds ratio (OR) of 10 for developing T2DM by age 32 [19].

It is unknown what the short-term risk of progression to T2DM is among adolescents with MetS; however, MetS appears to be a less specific marker than IGT of imminent risk of T2DM, given that MetS is classified in 8.6% percent of adolescents in the US [20] while the prevalence of T2DM among US adolescents in the SEARCH study was estimated at only 0.042% [21]. In addition, MetS exhibits racial/ethnic discrepancies that may limit its widespread use as a screening tool [22,23]. In particular, when using traditional MetS criteria based on the Adult Treatment Panel III (ATP-III), non-Hispanic black individuals are less likely to be classified as having MetS despite having more insulin resistance and more T2DM [24-26]. Because of these racial/ethnic differences, we have recently formulated a sex- and race/ethnicity-specific MetS severity score for use among adolescents [27]. This continuous estimate of MetS severity is a Z-score (range from negative infinity to positive infinity, with a mean of 0) is calculated based on an adolescent’s values for each of the components of MetS, with weighting of these components that is determined by confirmatory factor analysis of how these components cluster by sex- and racial/ethnicity and is unique to each subgroup [28]. This score appears less likely to exhibit racial ethnic differences in the association of MetS with surrogate markers of T2DM risk, such as elevated fasting insulin [27].

Our goal was to evaluate the potential for MetS to identify individuals with IGT, as an important precursor to T2DM. We utilized data from the National Health and Nutrition Evaluation Survey (NHANES) data with two hypotheses: 1) that traditional MetS would have a high sensitivity and specificity for predicting IGT and 2) that our sex- and race/ethnicity-specific risk score would exhibit improved sensitivity to identify adolescents with IGT. In doing so we hoped to clarify relationships between these markers of long-term risk T2DM risk among adolescents.

## Methods

Data were obtained from NHANES (1999–2010), a complex, multistage probability sample of the US population. These annual cross-sectional surveys are conducted by the National Center for Health Statistics (NCHS) of the Centers for Disease Control (CDC), with randomly-selected participants undergoing anthropometric and blood pressure measurements, answering questionnaires and undergoing phlebotomy (http://www.cdc.gov/nchs/nhanes.htm). The NCHS ethics review board reviewed and approved the survey and participants gave informed consent prior to participation.

Height, weight, BMI, WC, blood pressure (BP), and laboratory measures of triglycerides, HDL-C, and glucose were obtained using standardized protocols and calibrated equipment [29]. All blood samples used for analyses were obtained following a ≥8 hours fast.

A fasting oral glucose tolerance test was performed on a random sub-set of adolescent participants of NHANES. After their fasting glucose assessment, participants were given 1.75 g/kg of glucola to a maximum of 75 g. Two hours after this ingestion a second blood draw was obtained to assess 2-hour glucose level.

Data from non-Hispanic-white, non-Hispanic-black, or Hispanic (Mexican-American/other Hispanic) adolescents 12-19 y were analyzed. Children <12 y were excluded since fasting values for triglycerides and glucose were only obtained in participants ≥12 y. Participants with known diabetes (T1DM or T2DM) were excluded by eliminating those with self-reported diabetes and those on anti-diabetic medications. Participants were also excluded if they were pregnant or taking antihyperlipidemic medications as these are likely to alter lipid and glucose levels in a manner that may reflect baseline relationships between MetS and glucose tolerance. Individuals taking anti-hypertensive medication were classified as having hypertension.

### IGT and MetS classification

IGT was classified for a two-hour blood glucose of ≥140 mg/dL. MetS status was evaluated in two manners: 1) A commonly-used pediatric/adolescent adaptation of the Adult Treatment Panel III (ATP III) criteria [20,29]. Participants had to meet ≥3 of the following 5 criteria: concentration of triglycerides ≥110 mg/dL, HDL-C ≤40 mg/dL, WC ≥90th percentile for age/sex (or ATP III limit of 102 cm for males and 88 cm for females, whichever was lower) [12,30], glucose concentration ≥100 mg/dL, and systolic or diastolic BP ≥90th percentile (age, height, and sex-specific) [31]. Similarly, hypertension was defined as systolic or diastolic BP ≥90th percentile for age, height, and sex. 2). Using a pediatric-adolescent continuous metabolic syndrome severity Z-score [27]. This score is based on a factor analysis of the contributions of individual MetS components on a sex- and race/ethnicity-specific basis. The sex- and race/ethnicity-specific equations have been published previously [27] and are available as an online calculator (http://publichealth.hsc.wvu.edu/biostatistics/mets).

### Statistical analysis

Statistical significance was defined as a p-value < 0.05. Statistical analysis was performed using SAS (version 9.3, Cary, NC). Prevalence rates of MetS were calculated by glucose tolerance category, and compared via chi-square tests. The presence of insulin resistance was categorized based on an elevated fasting insulin ≥16.0 IU/mL, approximately the 95^th^ percentile among lean adolescents in NHANES [25] and used elsewhere previously [32-34]. Receiver operating characteristic (ROC) analysis was used to assess the ability of the sex- and race/ethnicity-specific MetS Z-score to discriminate IGT. Overall predictive performance was measured by the area under the curve (AUC) of the ROC curve, with AUC of 0.5 and 1.0 indicating no and perfect predictive ability, respectively. Sensitivities and specificities to predict the presence of IGT were compared between the traditional MetS classification and using cut-offs of the MetS severity Z-score. This includes Z-score cut-offs as follows: 1) 1.0 to approximate the prevalence of ATP-III MetS and 2) 0.75 assess the performance of a more liberal definition of MetS. These statistics were done on a sex and race/ethnicity-specific basis. All analyses, except for the ROC analysis, accounted for the survey design of NHANES in producing population-based estimates of basic descriptive statistics as well as prevalences, sensitivities, and specificities.

## Results

### Participant characteristics

We analyzed a study sample consisting of 1513 non-Hispanic-white, non-Hispanic-black and Hispanic adolescents age 12-19 y with data for all variables tested. Overall 4.8% of participants exhibited IGT while 7.92% were classified as having MetS (Table 1). Compared to those with normal glucose tolerance, those with IGT were older, had a higher BMI and a higher rate of ATP-III MetS classification. With the exception of fasting glucose, there were no significant differences in raw values of individual MetS components, though compared to adolescents with normal glucose tolerance, adolescents with IGT had a higher prevalence of elevated WC (21.3% vs. 44.7%, p < 0.01), high BP (7.4% vs. 21.4%, p < 0.01) and high fasting glucose (15.2% vs. 37.6%, p < 0.001). Adolescents with IGT had higher levels of fasting insulin (18.9 IU/mL vs. 11.8 IU/mL) and a higher prevalence of fasting insulin above 16.0 (the 95^th^ percentile among lead adolescents, 51.9 vs. 20.1, p < 0.0001). HbA1c values were similar between groups, as was the MetS Z-score.

**Table 1:** Participant characteristics

	**Overall**	**Normal glucose tolerance**	**Impaired glucose tolerance**	p-value*
N	1513	1441	72	
Mean (95% CI) Age	15.55 (15.38, 15.71)	15.60 (15.43, 15.76)	14.47 (13.75, 15.20)	0.0030
Percent (95% CI) Male	49.94 (45.06, 54.82)	50.33 (45.22, 55.44)	41.98 (22.68, 61.28)	0.4146
Mean (95% CI) BMI	23.87 (23.39, 24.34)	23.80 (23.34, 24.25)	25.35 (22.47, 28.24)	0.2797
Mean (95% CI) BMI Z-score	0.66 (0.57, 0.74)	0.65 (0.57, 0.73)	0.93 (0.37, 1.49)	0.3062
Percent (95% CI) with ATP-III MetS	7.92 (5.77, 10.06)	7.14 (5.00, 9.29)	23.74 (8.75, 38.73)	0.0009
MetS Components				
Waist Circumference				
Mean (95% CI)	82.21 (80.91, 83.50)	82.00 (80.72, 83.28)	86.45 (78.86, 94.05)	0.2486
Percent (95% CI) ≥ 90^th^ percentile	22.42 (19.01, 25.83)	21.34 (17.82, 24.85)	44.66 (27.32, 61.99)	0.0022
Triglycerides				
Mean (95% CI)	85.91 (81.65, 90.17)	84.42 (80.53, 88.32)	116.34 (78.83, 153.84)	0.0952
Percent (95% CI) ≥ 110	21.60 (18.53, 24.68)	21.01 (18.19, 23.82)	33.82 (15.83, 51.81)	0.0806
HDL				
Mean (95% CI)	53.00 (52.03, 53.97)	53.07 (52.08, 54.06)	51.54 (47.06, 56.03)	0.5079
Percent (95% CI) ≤ 40	11.46 (9.13, 13.78)	11.11 (8.82, 13.40)	18.63 (5.64, 31.61)	0.1541
Blood Pressure				
Mean Systolic (95% CI)	109.91 (108.87, 110.95)	109.83 (108.80, 110.87)	111.46 (105.90, 117.03)	0.5608
Percent (95% CI) ≥ 90^th^ percentile	8.01 (5.82, 10.20)	7.36 (9.57, 5.14)	21.42 (8.08, 34.76)	0.0025
Fasting Blood Glucose				
Mean (95% CI)	93.09 (92.36, 93.82)	92.90 (92.19, 93.62)	96.96 (93.86, 100.06)	0.0104
Percent (95% CI) ≥ 100	16.27 (13.41, 19.13)	15.23 (12.48, 17.99)	37.60 (21.08, 54.11)	0.0003
Mean (95% CI) HbA1c	5.26 (5.24, 5.28)	5.26 (5.23, 5.28)	5.32 (5.23, 5.40)	0.1938
Mean (95% CI) fasting insulin	12.17 (11.49, 12.85)	11.84 (11.29, 12.49)	18.90 (14.28, 23.51)	0.0033
Percent (95% CI) insulin > 16 IU/mL	21.62 (18.61, 24.64)	20.14 (17.26, 23.03)	51.89 (36.40, 67.37)	< 0.0001
Mean (95% CI) MetS Z-score	−0.02 (−0.09, 0.05)	−0.04 (−0.10, 0.03)	0.32 (−0.20, 0.83)	0.1703

*t-test comparing means of continuous variables; chi-square test comparing percentages of binary variables (both accounting for survey design).

### Ability of MetS classification to identify IGT

**Figure 1:**
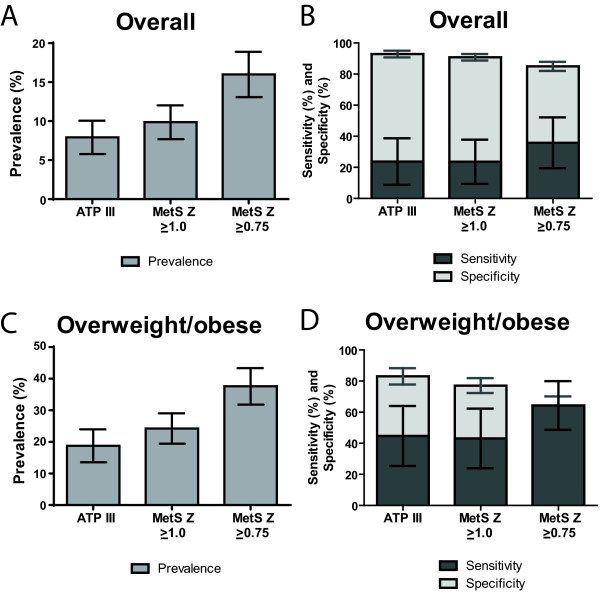
**Metabolic syndrome in adolescents: prevalence and sensitivity and specificity to identify impaired glucose tolerance (IGT). A** and **C**: Prevalence and 95% confidence intervals of MetS among all adolescents **(A)** and among only those overweight or obese **(C)**, using ATP-III MetS criteria and MetS Z-score with cut-offs of 1.0 and 0.75. **B** and **D**: Sensitivity (dark grey bars) and specificity (light grey bars) and 95% CI of MetS for identifying IGT among all adolescents **(B)** and among only those overweight or obese **(D)**.

Overall a classification of MetS exhibited poor sensitivity but reasonable specificity for the identification of IGT. Using traditional MetS criteria in the overall population, a classification of MetS had a sensitivity of 23.7% and specificity of 92.9% for detecting IGT, while using a MetS Z-score cut off of 1.0 (to yield similar MetS prevalence as found by traditional MetS criteria) had a sensitivity of 23.6% and specificity of 90.8% (Figure 1). The overall poor sensitivity was also noted among adolescents who were overweight/obese, for whom ATP-III MetS had a sensitivity of 44.7% and specificity of 83.0% for detecting IGT, while using a MetS Z-score cut off of 1.0 had a sensitivity of 43.1% and specificity of 77.1%. As expected, using a more liberal MetS Z-score cut-off of 0.75 yielded higher sensitivity values (35.8% overall and 64.3% among those overweight/obese) and lower specificity values (85.0% overall and 64.3% among those overweight). When evaluated by race/ethnicity, both MetS measures yielded lower sensitivity among non-Hispanic whites compared to non-Hispanic blacks and Hispanics (Table 2). Compared to ATP-III MetS, the MetS Z-score at a cut-off of 1.0 had lower sensitivity among non-Hispanic whites (11.2% vs. 17.4%) but higher sensitivity among non-Hispanic blacks (55.9% vs. 31.8%) and similar among Hispanics (37.1% vs. 34.1%).

**Table 2:** Sensitivity and specificity (and 95% confidence intervals) of metabolic syndrome to predict impaired glucose tolerance by racial/ethnic group among adolescents overall and among those who are overweight/obese

		**All adolescents**			**Adolescents BMI ≥ 85**^**th **^**percentile**		
		**Prevalence of “Mets”**	**Sensitivity**	**Specificity**	**Prevalence of “Mets”**	**Sensitivity**	**Specificity**
NHW	ATP-III MetS	7.43	17.36	93.01	18.57	43.97	82.74
		(4.46, 10.41)	(0.00, 35.00)	(90.03, 96.00)	(10.33, 26.80)	(9.21, 78.72)	(74.68, 90.79)
	MetS Z > 1.0	8.33	11.17	91.79	22.09	28.29	78.22
		(5.27, 11.40)	(0.00, 24.47)	(88.79, 94.79)	(14.28, 29.91)	(0, 58.82)	(70.65, 85.80)
	MetS Z > 0.75	14.48	23.63	85.92	36.64	59.85	64.55
		(10.18, 18.79)	(2.81, 44.44)	(81.60, 90.26)	(27.13, 46.15)	(27.78, 91.93)	(54.95, 74.14)
NHB	ATP-III MetS	4.40	31.82	96.55	9.91	34.33	91.99
		(2.10, 6.69)	(1.06, 62.58)	(94.64, 98.46)	(4.90, 14.92)	(1.92, 66.75)	(87.67, 96.32)
	MetS Z > 1.0	7.73	55.91	93.94	17.39	60.33	85.95
		(5.00, 10.46)	(24.42, 87.40)	(91.58, 96.31)	(11.89, 22.89)	(28.88, 91.78)	(9.33, 18.77)
	MetS Z > 0.75	13.18	69.32	88.76	29.90	74.80	74.59
		(8.14, 18.22)	(41.80, 96.83)	(84.18, 93.35)	(19.23, 38.71)	(48.27, 100)	(65.08, 84.10)
Hispanic	ATP-III MetS	12.49	34.06	89.12	26.13	51.33	76.68
		(8.65, 16.32)	(12.39, 55.73)	(85.82, 92.42)	(18.94, 33.32)	(27.67, 74.99)	(16.60, 30.04)
	MetS Z > 1.0	16.90	37.07	84.61	35.15	52.19	66.75
		(12.63, 21.16)	(15.71, 58.42)	(80.82, 88.39)	(27.67, 42.62)	(28.83, 75.53)	(59.65, 73.85)
	MetS Z > 0.75	23.49	48.17	78.35	46.73	64.02	55.20
		(18.60, 28.38)	(26.73, 69.60)	(73.82, 82.89)	(38.97, 54.49)	(42.70, 85.35)	(47.59, 62.80)

**Figure 2:**
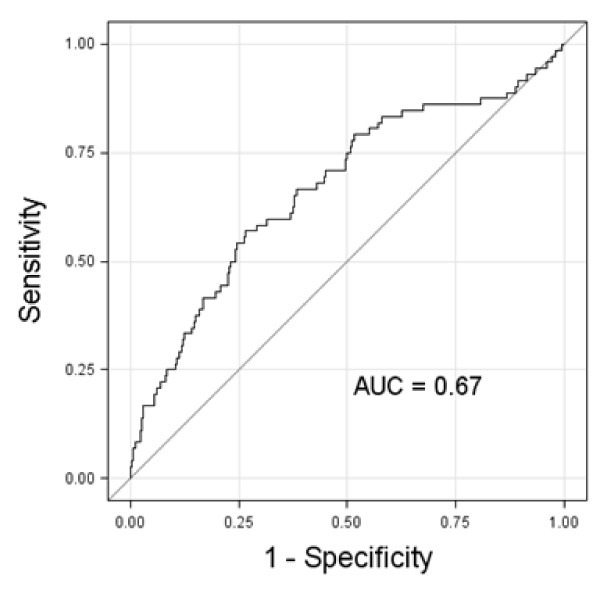
**ROC curve of metabolic syndrome Z-score for identifying impaired glucose tolerance (IGT).** ROC curve among all adolescents using MetS Z-score. AUC = area under curve.

ROC curve analysis of the race/ethnicity-specific Adolescent MetS Z-score to identify participants with IGT revealed an area-under-the-curve of 0.67 among the overall group (Figure 2). When evaluated among only those adolescents who were overweight/obese this area-under-the-curve was 0.64.

## Discussion

In this sample of adolescents who received an OGTT as part of NHANES, we were surprised to find a relatively poor association between two important markers of risk for T2DM: MetS and IGT. While these processes are clearly linked—seen in a 3-fold higher prevalence of MetS among adolescents with IGT—we found that the majority of cases of IGT were not associated with MetS, either using traditional ATP-III criteria or using a sex- and race/ethnicity-specific linear MetS Z-score. This low prevalence of MetS in IGT was true both in the overall population and in a sub-set of adolescents who were overweight/obese and thus more likely to experience the processes underlying MetS, such as adipocyte dysfunction and oxidative stress. Altogether, this lower-than-anticipated association between MetS and IGT may implicate a predominance of non-MetS causes of IGT in adolescents.

The low association of MetS with IGT in adolescents is likely related to the complex physiology of glucose control. Elevations in BG following a glucose challenge are influenced by insulin release and tissue responsiveness to insulin [17]. Insulin release following glucose ingestion occurs in two phases: an initial spike (the first phase, blunting of which is an early occurrence in the pathophysiology of T2DM) and a second more gradual rise that can be heightened in T2DM but remains inadequate to lower BG [35]. Insulin secretion is influenced by underlying genes affecting beta cell function (which constitute the largest group of genes implicated in T2DM pathogenesis in large-scale evaluations [18]) but can be suppressed further by elevations in lipids [36,37] and glucose itself [38], as well as dysfunction of other hormones including the incretin GLP-1 [17]. Insulin resistance can contribute to BG elevations by necessitating that beta cells secrete higher amounts of insulin to mediate sufficient glucose disposal. Once the minimum threshold of insulin level for maintenance of BG levels is exceeded, post-prandial BG begins to rise. The combination of inadequate insulin secretion and insulin resistance is the primary cause of IGT and subsequent diabetes in adults [39-41].

Among children and adolescents, the major cause of IGT is unknown, while the major cause of diabetes remains isolated defects in insulin production as seen following auto-immune beta cell destruction in T1DM, with a prevalence of 0.23% among adolescents compared to a prevalence of 0.042% for T2DM in this age range [21]. Limitations in insulin release are also seen in monogenic forms of diabetes such as the group of genes comprising MODY, also with a low overall prevalence at approximately 1% of pediatric diabetes cases [42,43]. Whereas insulin resistance and MetS are more prevalent in overweight/obese children and adolescents [44] (contributing to an improved sensitivity of MetS for IGT identification among overweight/obese adolescents, Table 2), primary defects in insulin release such as T1DM and MODY would be expected to be present across the weight spectrum in childhood. T1DM itself, if poorly controlled, is clearly associated with abnormalities in MetS components, particularly hypertriglyceridemia and low HDL [45], further complicating the potential relationships between MetS and IGT. We excluded participants with known diabetes from our analysis, but it is possible that a small percentage of the adolescents in our sample had early, undiagnosed T1DM or MODY [10]. Nevertheless, the proportion of all participants in our sample with non-MetS IGT (3.6%) far exceeded the expected number of cases of undiagnosed T1DM and MODY (which together have an incidence of 0.022% per year in childhood [21,46]), potentially suggesting a high prevalence of other limitations of insulin secretion, including polygenic defects in beta-cell function that have been implicated in T2DM in adults [18,47].

We hypothesized that one limitation in the ability of MetS to identify individuals with IGT was due to variation in the diagnostic accuracy of MetS criteria by racial/ethnic group [22-26]. Because of this, we performed our analysis using both a common adolescent adaptation of ATP-III MetS criteria [20,29], as well as a sex- and race/ethnicity-specific MetS severity score [27]. This severity score has the potential to have cut-off levels adjusted based on outcomes-based data (which we currently lack) or based on a desire to identify higher numbers of adolescents at risk. In using this score we first tested a cut-off level of 1 standard deviation above the mean, which provides prevalence of MetS similar to that determined by ATP-III criteria—and which produced sensitivity values for IGT prediction similar to ATP-III criteria. We then tested a more liberal cut-off of 0.75 standard deviations above the mean, exhibiting increased sensitivity but worsened specificity compared to ATP-III-based criteria. This type of approach could be used to improve identification of adolescents at increased risk for long-term diseases associated with MetS—which could be important since improved tools for risk detection are badly needed to target interventions to help avert disease progression [23].

The MetS Z-score exhibited a differential response in IGT prediction by racial/ethnic group, with the score overall exhibiting worsened sensitivity among non-Hispanic white adolescents (using a MetS Z-score cut-off 1.0, sensitivity was 11.2% vs. 17.4% for ATP-III MetS) but an improved sensitivity among non-Hispanic blacks (55.9% vs. 31.8%). This improvement in sensitivity among non-Hispanic black adolescents may not be surprising, since traditional MetS criteria utilize population-based cut-off values for the individual components and do not take into account that non-Hispanic-black adolescents have lower baseline levels of triglycerides and are less likely to exhibit abnormalities in triglycerides or HDL despite having more insulin resistance and higher rates of diabetes [24-26]. These inter-ethnicity differences are what stimulated our formulation of the sex- and race/ethnicity-specific MetS Z-score in the first place. Overall, the MetS Z-score appeared to work best in the identification of non-Hispanic black adolescents with IGT, though we were limited in many of our comparisons between racial/ethnic groups by the small sample size of adolescents in NHANES who underwent OGTT’s and by the overall lower prevalence of IGT.

However, while we noted an increased sensitivity using a lower cur-off of the race/ethnicity-specific MetS Z-score, the presence of MetS overall was a poor screening test to identify adolescents with IGT. Acceptable degrees of sensitivity and specificity in a screening test depend on the importance of the outcome being screened for and the downside to missing detection of that outcome. In this case, the specificity of these tests was overall reasonable clinically, 78-97% among all adolescents. However, the low sensitivity and thus number of non-MetS cases of IGT reflects the potential for false reassurance regarding the risk of IGT in an adolescent based on the absence of MetS.

Interestingly, there appeared to be a sizable number of cases of elevated fasting insulin levels (as an estimate of insulin resistance) in this sample that were not identified by ATP-III MetS or our MetS Z-score. Among adolescents with IGT there was a higher prevalence of elevated fasting insulin—present in 52% of adolescents with IGT—than of ATP-III MetS (24%) or any of the individual components of MetS (with prevalences of 19-45%). There is a clear difficulty in using measures of fasting insulin as an estimate of insulin resistance in settings of glucose excursions, since the mere elevation of BG implies a limitation in secretion of adequate amounts of insulin to normalize BG. Additionally, while fasting insulin correlates highly with other surrogate markers of insulin resistance (HOMA-IR, QUICKI), it clearly lacks the precision of more robust measures of insulin resistance, such as an insulin clamp [44,48]. Thus, our measure of those with elevated insulin levels (fasting insulin above 16.0 IU/mL, approximately the 95^th^ percentile among lean adolescents in NHANES [25] and used elsewhere [32-34]) may not reflect the full number of participants with insulin resistance. Overall, however, there were 48% of children with IGT who did not exhibit elevations in fasting insulin, again suggesting a high prevalence of IGT unrelated to insulin resistance.

This study had multiple limitations, including the cross-sectional design of NHANES, which limits any conclusions regarding causality. In addition, while NHANES often represents a powerful study, there was only a small subset of adolescents who underwent the OGTT. Finally, we lacked important additional information such as antibodies associated with T1DM as well as genetic information on participants.

In conclusion, we found that the presence of MetS and elevated fasting insulin in adolescents had a poor correlation with IGT, an important precursor T2DM as well as a potential finding during the short pre-symptomatic phase of T1DM. This lack of overlap between MetS and IGT may indicate that assessment of MetS is not likely to be a good indicator of which adolescents to screen using an OGTT. These data further underscore the need for further research to assess for other potential contributors to IGT, including T1DM, MODY and polymorphisms associated with poorer beta-cell function. Practitioners should keep in mind other potential causes of IGT, even when evaluating obese adolescents with IGT.

## Abbreviations

ATP-III: Adult treatment panel III; BG: Blood glucose; HDL: High density lipoprotein; IGT: Impaired glucose tolerance; MetS: Metabolic syndrome; MODY: Maturity onset diabetes of the young; NHANES: National health and nutrition examination survey; OGTT: Oral glucose tolerance test; T1DM: Type 1 diabetes mellitus; T2DM: Type 2 diabetes mellitus.

## Competing interests

The authors have no conflicts of interest to declare.

## Authors' contributions

MDD was involved in the design, interpretation and write-up of the study and approved of the final manuscript as submitted. MJG was involved in the design, analysis, interpretation and write-up of the study and approved of the final manuscript as submitted.
